# 5-Methyl-2,4-bis­(methyl­sulfan­yl)tricyclo­[6.2.1.0^2,7^]undeca-4,9-diene-3,6-dione^1^

**DOI:** 10.1107/S1600536810015710

**Published:** 2010-05-08

**Authors:** Andreas A. von Richthofen, José E. P. Cardoso Filho, Liliana Marzorati, Julio Zukerman-Schpector, Edward R. T. Tiekink, Claudio Di Vitta

**Affiliations:** a Chemistry Institute of the University of São Paulo, Av. Prof. Lineu Prestes 748, 05508-000, São Paulo, SP, Brazil; b Department of Chemistry, Universidade Federal de São Carlos, 13565-905 São Carlos, SP, Brazil; c Department of Chemistry, University of Malaya, 50603 Kuala Lumpur, Malaysia

## Abstract

The structure analysis of the title compound, C_14_H_16_O_2_S_2_, shows the SMe and H atoms of the bond linking the six-membered rings to be *syn* and also to be *syn* to the bridgehead –CH_2_– group. Each of the five-membered rings adopts an envelope conformation at the bridgehead –CH_2_– group. The dione-substituted ring adopts a folded conformation about the 1,4-C⋯C vector, with the ketone groups lying to one side. The cyclo­hexene ring adopts a boat conformation.

## Related literature

For background to reactions of toluquinone-cyclo­penta­diene Diels–Alder adducts epoxides with nucleophiles under heterogeneous conditions, see: von Richthofen *et al.* (2010 ▶). For conformational analysis, see: Cremer & Pople (1975 ▶).
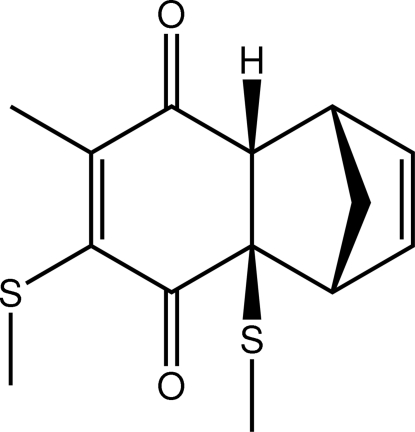


## Experimental

### 

#### Crystal data

C_14_H_16_O_2_S_2_
*M* *_r_* = 280.39
Monoclinic, 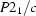

*a* = 9.1109 (11) Å
*b* = 17.3009 (19) Å
*c* = 9.3746 (11) Å
β = 115.916 (2)°
*V* = 1329.1 (3) Å^3^
*Z* = 4
Mo *K*α radiation
μ = 0.39 mm^−1^
*T* = 98 K
0.28 × 0.18 × 0.15 mm

#### Data collection

Rigaku AFC12/SATURN724 diffractometer
Absorption correction: multi-scan (*ABSCOR*; Higashi, 1995 ▶) *T*_min_ = 0.887, *T*_max_ = 1
10394 measured reflections
3044 independent reflections
2974 reflections with *I* > 2σ(*I*)
*R*_int_ = 0.021

#### Refinement

*R*[*F* ^2^ > 2σ(*F* ^2^)] = 0.035
*wR*(*F* ^2^) = 0.092
*S* = 1.02
3044 reflections
164 parameters
H-atom parameters constrained
Δρ_max_ = 0.53 e Å^−3^
Δρ_min_ = −0.36 e Å^−3^

### 

Data collection: *CrystalClear* (Rigaku/MSC, 2005 ▶); cell refinement: *CrystalClear*; data reduction: *CrystalClear*; program(s) used to solve structure: *PATTY* in *DIRDIF92* (Beurskens *et al.*, 1992 ▶); program(s) used to refine structure: *SHELXL97* (Sheldrick, 2008 ▶); molecular graphics: *ORTEP-3* (Farrugia, 1997 ▶); software used to prepare material for publication: *publCIF* (Westrip, 2010 ▶).

## Supplementary Material

Crystal structure: contains datablocks global, I. DOI: 10.1107/S1600536810015710/hg2678sup1.cif

Structure factors: contains datablocks I. DOI: 10.1107/S1600536810015710/hg2678Isup2.hkl

Additional supplementary materials:  crystallographic information; 3D view; checkCIF report

## Figures and Tables

**Fig. 1.**
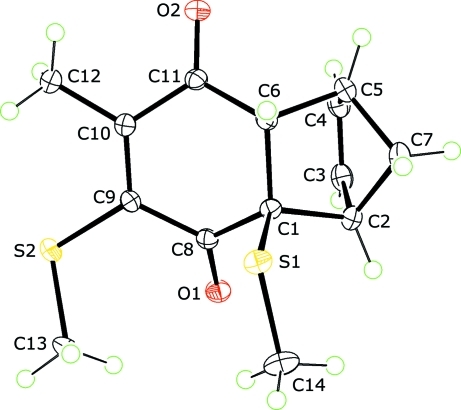
The molecular structure of (I) showing the atom-labelling scheme and displacement ellipsoids at the 50% probability level.

## supplementary crystallographic information

### Comment

The structure of the title compound, (I), was investigated as a part of a study into the reactions of toluquinone-cyclopentadiene Diels-Alder adducts epoxides with nucleophiles under heterogeneous conditions (von Richthofen *et al.*, 2010). The most important feature of the molecular structure, Fig. 1, is the *syn* relationship between the bridgehead-C7, S1 and H6 atoms; the oxo groups and double bond of the hexene residue lie to the opposite side of the molecule to these atoms. The conformation of each of the five-membered rings in (I) is an envelope on C7; the ring puckering parameters (Cremer & Pople, 1975) are Q_2_ = 0.6188 (18) Å and φ_2_ = 252.91 (16) ° for C1,C2,C5–C7, and Q_2_ = 0.5393 (18) Å and φ_2_ = 323.49 (19) ° for C2–C5,C6. The cyclohexene ring, C1–C6, adopts a boat form with ring-puckering parameters of q_2_ = 0.9782 (17) Å, q_3_ = 0.0101 (17) Å, θ = 89.41 (10) °, and φ_2_ = 59.60 (10) °. Finally, the C1,C6,C8–C11 dione-substituted ring adopts a folded conformation about the C8–C11 vector. The C1,C6,C9,C10 atoms define a plane [r.m.s. deviation = 0.0069 Å] with the C8 and C11 atoms lying 0.3859 (20) and 0.3099 (21) Å out of this plane, respectively; the O1 and O2 atoms lie even further out of the plane, i.e. 0.950 (3) and -0.743 (3) Å, respectively. No specific intermolecular interactions are noted in the crystal packing.

### Experimental

The preparation and characterisation is as described in the literature (von Richthofen *et al.*, 2010). The crystals were obtained by slow evaporation at 253 K from a 6:1 solution of *n*-hexane:ethyl acetate.

### Refinement

The H atoms were geometrically placed (C—H = 0.93–0.98 Å) and refined as riding with *U*_iso_(H) = 1.2-1.5*U*_eq_(C).

### Crystal data

**Table:**

C_14_H_16_O_2_S_2_	*F*(000) = 592
*M**_r_* = 280.39	*D*_x_ = 1.401 Mg m^−^^3^
Monoclinic, *P*2_1_/*c*	Mo *K*α radiation, λ = 0.71073 Å
Hall symbol: -P 2ybc	Cell parameters from 5730 reflections
*a* = 9.1109 (11) Å	θ = 2.4–40.4°
*b* = 17.3009 (19) Å	µ = 0.39 mm^−^^1^
*c* = 9.3746 (11) Å	*T* = 98 K
β = 115.916 (2)°	Block, pale-yellow
*V* = 1329.1 (3) Å^3^	0.28 × 0.18 × 0.15 mm
*Z* = 4	

### Data collection

**Table:**

Rigaku AFC12K/SATURN724 diffractometer	3044 independent reflections
Radiation source: fine-focus sealed tube	2974 reflections with *I* > 2σ(*I*)
graphite	*R*_int_ = 0.021
ω scans	θ_max_ = 27.5°, θ_min_ = 2.4°
Absorption correction: multi-scan (*ABSCOR*; Higashi, 1995)	*h* = −11→11
*T*_min_ = 0.887, *T*_max_ = 1	*k* = −22→16
10394 measured reflections	*l* = −12→12

### Refinement

**Table:**

Refinement on *F*^2^	Primary atom site location: structure-invariant direct methods
Least-squares matrix: full	Secondary atom site location: difference Fourier map
*R*[*F*^2^ > 2σ(*F*^2^)] = 0.035	Hydrogen site location: inferred from neighbouring sites
*wR*(*F*^2^) = 0.092	H-atom parameters constrained
*S* = 1.02	*w* = 1/[σ^2^(*F*_o_^2^) + (0.0478*P*)^2^ + 1.0842*P*] where *P* = (*F*_o_^2^ + 2*F*_c_^2^)/3
3044 reflections	(Δ/σ)_max_ < 0.001
164 parameters	Δρ_max_ = 0.53 e Å^−^^3^
0 restraints	Δρ_min_ = −0.36 e Å^−^^3^

### Special details

**Table:**

Geometry. All esds (except the esd in the dihedral angle between two l.s. planes) are estimated using the full covariance matrix. The cell esds are taken into account individually in the estimation of esds in distances, angles and torsion angles; correlations between esds in cell parameters are only used when they are defined by crystal symmetry. An approximate (isotropic) treatment of cell esds is used for estimating esds involving l.s. planes.
Refinement. Refinement of *F*^2^ against ALL reflections. The weighted *R*-factor wR and goodness of fit *S* are based on *F*^2^, conventional *R*-factors *R* are based on *F*, with *F* set to zero for negative *F*^2^. The threshold expression of *F*^2^ > σ(*F*^2^) is used only for calculating *R*-factors(gt) etc. and is not relevant to the choice of reflections for refinement. *R*-factors based on *F*^2^ are statistically about twice as large as those based on *F*, and *R*- factors based on ALL data will be even larger.

### Fractional atomic coordinates and isotropic or equivalent isotropic displacement parameters (Å^2^)

**Table:**

	*x*	*y*	*z*	*U*_iso_*/*U*_eq_	
S1	0.09559 (4)	0.19478 (2)	0.09496 (4)	0.01770 (11)	
S2	0.59801 (5)	0.11808 (2)	0.12851 (4)	0.01903 (11)	
O1	0.29429 (13)	0.03034 (6)	0.10133 (13)	0.0187 (2)	
O2	0.54427 (14)	0.17201 (7)	0.62713 (13)	0.0217 (2)	
C1	0.20518 (17)	0.11982 (8)	0.24056 (16)	0.0131 (3)	
C2	0.09479 (18)	0.05593 (8)	0.26238 (18)	0.0166 (3)	
H2	0.0093	0.0341	0.1651	0.020*	
C3	0.21532 (18)	−0.00100 (9)	0.37784 (19)	0.0186 (3)	
H3	0.2376	−0.0504	0.3534	0.022*	
C4	0.28332 (19)	0.03223 (9)	0.52005 (19)	0.0192 (3)	
H4	0.3609	0.0102	0.6129	0.023*	
C5	0.21127 (18)	0.11264 (9)	0.50320 (18)	0.0176 (3)	
H5	0.2198	0.1368	0.6011	0.021*	
C6	0.28355 (17)	0.15956 (8)	0.40699 (17)	0.0143 (3)	
H6	0.2405	0.2123	0.3945	0.017*	
C7	0.03709 (19)	0.09835 (9)	0.37360 (19)	0.0201 (3)	
H7A	−0.0213	0.1458	0.3281	0.024*	
H7B	−0.0269	0.0657	0.4092	0.024*	
C8	0.32628 (17)	0.08718 (8)	0.18602 (16)	0.0136 (3)	
C9	0.48477 (17)	0.12970 (8)	0.23722 (17)	0.0136 (3)	
C10	0.55309 (17)	0.16556 (8)	0.38032 (17)	0.0142 (3)	
C11	0.46773 (17)	0.16467 (8)	0.48354 (17)	0.0146 (3)	
C12	0.71871 (18)	0.20233 (9)	0.44683 (18)	0.0183 (3)	
H12A	0.8009	0.1631	0.4907	0.027*	
H12B	0.7286	0.2383	0.5285	0.027*	
H12C	0.7327	0.2291	0.3639	0.027*	
C13	0.4452 (2)	0.11235 (10)	−0.07521 (18)	0.0232 (3)	
H13A	0.4978	0.1060	−0.1436	0.035*	
H13B	0.3819	0.1590	−0.1028	0.035*	
H13C	0.3749	0.0690	−0.0873	0.035*	
C14	−0.0177 (2)	0.13994 (10)	−0.08395 (19)	0.0257 (3)	
H14A	−0.0803	0.1746	−0.1689	0.039*	
H14B	−0.0898	0.1047	−0.0668	0.039*	
H14C	0.0568	0.1114	−0.1112	0.039*	

### Atomic displacement parameters (Å^2^)

**Table:**

	*U*^11^	*U*^22^	*U*^33^	*U*^12^	*U*^13^	*U*^23^
S1	0.01849 (19)	0.01411 (19)	0.01702 (18)	0.00254 (13)	0.00454 (15)	0.00180 (13)
S2	0.0191 (2)	0.0247 (2)	0.01740 (19)	−0.00085 (14)	0.01171 (15)	−0.00128 (14)
O1	0.0216 (5)	0.0166 (5)	0.0186 (5)	−0.0013 (4)	0.0093 (4)	−0.0041 (4)
O2	0.0207 (6)	0.0295 (6)	0.0137 (5)	−0.0047 (4)	0.0064 (4)	−0.0031 (4)
C1	0.0132 (6)	0.0120 (6)	0.0127 (6)	0.0011 (5)	0.0045 (5)	0.0013 (5)
C2	0.0148 (7)	0.0163 (7)	0.0189 (7)	−0.0022 (5)	0.0075 (6)	0.0001 (5)
C3	0.0212 (7)	0.0136 (7)	0.0245 (7)	0.0008 (5)	0.0133 (6)	0.0039 (6)
C4	0.0212 (7)	0.0180 (7)	0.0210 (7)	0.0010 (6)	0.0116 (6)	0.0046 (6)
C5	0.0186 (7)	0.0195 (7)	0.0178 (7)	−0.0001 (5)	0.0109 (6)	0.0007 (6)
C6	0.0160 (7)	0.0146 (6)	0.0140 (6)	−0.0005 (5)	0.0079 (5)	−0.0011 (5)
C7	0.0175 (7)	0.0208 (7)	0.0254 (8)	0.0011 (6)	0.0126 (6)	0.0026 (6)
C8	0.0154 (7)	0.0130 (6)	0.0119 (6)	0.0011 (5)	0.0054 (5)	0.0026 (5)
C9	0.0149 (6)	0.0133 (6)	0.0141 (6)	0.0010 (5)	0.0076 (5)	0.0014 (5)
C10	0.0143 (6)	0.0127 (6)	0.0155 (6)	0.0006 (5)	0.0065 (5)	0.0011 (5)
C11	0.0172 (7)	0.0122 (6)	0.0145 (6)	−0.0014 (5)	0.0070 (6)	−0.0014 (5)
C12	0.0156 (7)	0.0204 (7)	0.0191 (7)	−0.0034 (5)	0.0078 (6)	−0.0018 (6)
C13	0.0317 (9)	0.0263 (8)	0.0146 (7)	0.0017 (7)	0.0130 (7)	0.0011 (6)
C14	0.0246 (8)	0.0248 (8)	0.0178 (7)	0.0020 (6)	0.0001 (6)	−0.0012 (6)

### Geometric parameters (Å, °)

**Table:**

S1—C14	1.8071 (17)	C5—H5	0.9800
S1—C1	1.8304 (14)	C6—C11	1.512 (2)
S2—C9	1.7500 (14)	C6—H6	0.9800
S2—C13	1.8086 (17)	C7—H7A	0.9700
O1—C8	1.2167 (18)	C7—H7B	0.9700
O2—C11	1.2223 (18)	C8—C9	1.5003 (19)
C1—C8	1.5135 (19)	C9—C10	1.357 (2)
C1—C6	1.5630 (19)	C10—C11	1.4830 (19)
C1—C2	1.5668 (19)	C10—C12	1.4994 (19)
C2—C3	1.520 (2)	C12—H12A	0.9600
C2—C7	1.543 (2)	C12—H12B	0.9600
C2—H2	0.9800	C12—H12C	0.9600
C3—C4	1.330 (2)	C13—H13A	0.9600
C3—H3	0.9300	C13—H13B	0.9600
C4—C5	1.517 (2)	C13—H13C	0.9600
C4—H4	0.9300	C14—H14A	0.9600
C5—C7	1.540 (2)	C14—H14B	0.9600
C5—C6	1.557 (2)	C14—H14C	0.9600
			
C14—S1—C1	102.96 (7)	C2—C7—H7A	112.9
C9—S2—C13	104.11 (7)	C5—C7—H7B	112.9
C8—C1—C6	114.76 (11)	C2—C7—H7B	112.9
C8—C1—C2	112.65 (11)	H7A—C7—H7B	110.3
C6—C1—C2	102.67 (11)	O1—C8—C9	121.85 (13)
C8—C1—S1	104.66 (9)	O1—C8—C1	121.33 (13)
C6—C1—S1	107.16 (9)	C9—C8—C1	116.80 (12)
C2—C1—S1	115.16 (10)	C10—C9—C8	120.12 (13)
C3—C2—C7	100.40 (12)	C10—C9—S2	119.71 (11)
C3—C2—C1	104.19 (11)	C8—C9—S2	119.17 (10)
C7—C2—C1	100.39 (11)	C9—C10—C11	119.70 (13)
C3—C2—H2	116.5	C9—C10—C12	123.26 (13)
C7—C2—H2	116.5	C11—C10—C12	116.91 (12)
C1—C2—H2	116.5	O2—C11—C10	120.53 (13)
C4—C3—C2	107.98 (13)	O2—C11—C6	120.63 (13)
C4—C3—H3	126.0	C10—C11—C6	118.73 (12)
C2—C3—H3	126.0	C10—C12—H12A	109.5
C3—C4—C5	107.54 (14)	C10—C12—H12B	109.5
C3—C4—H4	126.2	H12A—C12—H12B	109.5
C5—C4—H4	126.2	C10—C12—H12C	109.5
C4—C5—C7	100.65 (12)	H12A—C12—H12C	109.5
C4—C5—C6	105.37 (12)	H12B—C12—H12C	109.5
C7—C5—C6	100.23 (12)	S2—C13—H13A	109.5
C4—C5—H5	116.1	S2—C13—H13B	109.5
C7—C5—H5	116.1	H13A—C13—H13B	109.5
C6—C5—H5	116.1	S2—C13—H13C	109.5
C11—C6—C5	114.71 (12)	H13A—C13—H13C	109.5
C11—C6—C1	115.31 (11)	H13B—C13—H13C	109.5
C5—C6—C1	102.97 (11)	S1—C14—H14A	109.5
C11—C6—H6	107.8	S1—C14—H14B	109.5
C5—C6—H6	107.8	H14A—C14—H14B	109.5
C1—C6—H6	107.8	S1—C14—H14C	109.5
C5—C7—C2	94.11 (11)	H14A—C14—H14C	109.5
C5—C7—H7A	112.9	H14B—C14—H14C	109.5
			
C14—S1—C1—C8	67.48 (11)	C3—C2—C7—C5	48.84 (12)
C14—S1—C1—C6	−170.26 (10)	C1—C2—C7—C5	−57.84 (12)
C14—S1—C1—C2	−56.77 (12)	C6—C1—C8—O1	148.85 (13)
C8—C1—C2—C3	55.85 (15)	C2—C1—C8—O1	31.83 (18)
C6—C1—C2—C3	−68.13 (13)	S1—C1—C8—O1	−93.99 (14)
S1—C1—C2—C3	175.78 (10)	C6—C1—C8—C9	−33.00 (17)
C8—C1—C2—C7	159.48 (12)	C2—C1—C8—C9	−150.01 (12)
C6—C1—C2—C7	35.50 (13)	S1—C1—C8—C9	84.16 (12)
S1—C1—C2—C7	−80.59 (12)	O1—C8—C9—C10	−148.32 (14)
C7—C2—C3—C4	−32.22 (15)	C1—C8—C9—C10	33.53 (19)
C1—C2—C3—C4	71.40 (15)	O1—C8—C9—S2	20.23 (19)
C2—C3—C4—C5	−0.54 (16)	C1—C8—C9—S2	−157.91 (10)
C3—C4—C5—C7	33.23 (15)	C13—S2—C9—C10	−153.96 (12)
C3—C4—C5—C6	−70.61 (15)	C13—S2—C9—C8	37.43 (13)
C4—C5—C6—C11	−59.34 (15)	C8—C9—C10—C11	−2.0 (2)
C7—C5—C6—C11	−163.48 (12)	S2—C9—C10—C11	−170.46 (10)
C4—C5—C6—C1	66.75 (14)	C8—C9—C10—C12	173.67 (13)
C7—C5—C6—C1	−37.39 (13)	S2—C9—C10—C12	5.2 (2)
C8—C1—C6—C11	4.20 (17)	C9—C10—C11—O2	155.13 (14)
C2—C1—C6—C11	126.78 (12)	C12—C10—C11—O2	−20.8 (2)
S1—C1—C6—C11	−111.53 (11)	C9—C10—C11—C6	−28.7 (2)
C8—C1—C6—C5	−121.50 (12)	C12—C10—C11—C6	155.42 (13)
C2—C1—C6—C5	1.07 (13)	C5—C6—C11—O2	−38.17 (19)
S1—C1—C6—C5	122.77 (10)	C1—C6—C11—O2	−157.58 (14)
C4—C5—C7—C2	−49.33 (13)	C5—C6—C11—C10	145.63 (13)
C6—C5—C7—C2	58.61 (12)	C1—C6—C11—C10	26.22 (18)
